# Factors influencing length of stay and costs in inpatient cases of human brucellosis as the primary diagnosis over a decade in Beijing, China

**DOI:** 10.3389/fpubh.2024.1347693

**Published:** 2024-05-15

**Authors:** Xiaolong Ma, Wenqing Wang, Qiong Wu, Chao Zheng, Jianchao Liu, Huajuan Bai, Tianyi Zhang, Lin Li, Lihua Liu

**Affiliations:** ^1^Executive Office, Medical Service Department of PLA General Hospital, Beijing, China; ^2^Hospital Management Institute, Department of Innovative Medical Research, Chinese PLA General Hospital, Beijing, China; ^3^Graduate School of PLA General Hospital, Chinese PLA General Hospital, Beijing, China; ^4^Institute of Medical Information, Chinese Academy of Medical Sciences & Peking Union Medical College, Beijing, China

**Keywords:** human brucellosis, length of stay, total costs, utilization categories, infection sites

## Abstract

**Aims:**

In the year 2021, human brucellosis ranked fifth in terms of the number of cases among all statutorily notifiable infectious diseases in China, thus remaining a significant concern for public health. This study aims to provide insights into the financial burden of human brucellosis by examining hospital stays and associated costs for affected individuals.

**Methods:**

In this retrospective study, we gathered updated data from 467 inpatient cases primarily diagnosed with human brucellosis at eight major tertiary hospitals in Beijing, China, spanning from 2013 to 2023. To comprehensively explore the economic impact on individuals, we not only analyzed the duration of hospital stays and total costs but also examined various charge types, including drug, lab test, medical imaging, medical treatment, surgical procedures, medical supplies and consumables, inpatient bed care, nursing services, and other services costs. Statistical analysis was employed to compare differences among gender, age, ethnicity, type of health insurance, condition at admission, comorbidity index, the performance of surgery, and the site of infection.

**Results:**

Both the length of stay and total cost exhibited significant variations among insurance, surgery, and infection site groups. Utilization categories demonstrated significant differences between patients who underwent surgery and those who did not, as well as across different infection sites. Furthermore, multiple linear regression analysis revealed that the condition at admission, Elixhauser comorbidity index, infection site, and surgery influenced both hospital stay and total cost. In addition, age and insurance type were associated with total costs.

**Conclusion:**

By delving into various utilization categories, we have addressed a significant gap in the literature. Our findings provide valuable insights for optimizing the allocation and management of health resources based on the influencing factors identified in this study.

## 1 Introduction

Human brucellosis, caused by various species of Brucella bacteria, remains a significant global public health concern, with a notably pronounced impact in China (1). This infectious zoonotic disease is primarily transmitted through the consumption of contaminated dairy products or direct contact with infected animals, affecting various body systems and some specific sites such as spines, bones, joints, and the central nervous system. People infected with Human brucellosis typically manifests fever, weakness, sweating, chills, myalgia, spinal symptoms and so on. If the disease is not diagnosed and treated promptly, it may become chronic and persist for years, leading to complications such as osteoarticular, hepatobiliary, cardiovascular, and central nervous system diseases (1). Consequently, it poses a substantial health and economic burden (2).

China has experienced a steady growth in the incidence of human brucellosis cases (3). According to Chinese Center for Disease Control and Prevention, the number of cases increased from 47,425 in year 2020 to 69,767 in year 2021, and the peak season of human brucellosis is from March to August. About 95.5% of the total cases were centralized in northern China and 31.8% were in Inner Mongolia which is of advanced livestock farming (4). People frequently contact with domestic animals are potential high-risk groups, such as herdsmen, butchers, and veterinarians. The impact extends beyond public health, with profound socioeconomic repercussions due to healthcare costs, particularly the expenses associated with drugs and medications for treating human brucellosis. This constitutes a significant portion of the overall healthcare expenditure (3). Additionally, the length of hospital stay (LOS) directly influences both treatment costs and patient well-being. Examining the hospitalization period is crucial for optimizing the healthcare system, enhancing patient outcomes, and alleviating the strain on medical facilities (5). Furthermore, it can guide future policies aimed at minimizing the impact of human brucellosis on the Chinese healthcare system.

Existing research on the healthcare cost of human brucellosis in China may have limitations, including outdated data or a lack of a comprehensive analysis of various utilization categories (6, 7). To address these gaps, this study analyzed the healthcare costs of patients diagnosed with human brucellosis from eight large tertiary hospitals in Beijing from 2013 to 2023. The objective is to provide an up-to-date assessment of the factors influencing the length of stay and disease.

## 2 Materials and methods

### 2.1 Data source

**Figure 1:**
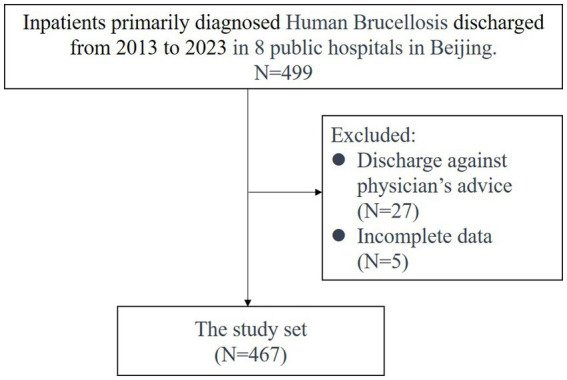
Flowchart of participant enrollment in the study set.

The data utilized in this retrospective observational study were obtained from the Hospital Information System of eight prominent tertiary hospitals in Beijing, which also encompassed an infectious disease hospital. The study incorporated a total of 499 patients discharged between January 1, 2013, and August 31, 2023, with a primary diagnosis of human brucellosis, as confirmed by the ICD code A23.9. Thirty-two inpatient cases were excluded from the analysis; among these, 27 participants were discharged against medical advice, while five cases had incomplete data. Although these five cases had total cost, the key utilization categories including drug, lab test, medical imaging, medical treatment, medical supplies and consumables, and inpatient bed care were missing. Ultimately, the final analysis included 467 patients. The study’s flow chart, outlining patient inclusion and exclusion criteria, is depicted in Figure 1. These patients hailed from various regions across China, with the majority originating from Hebei Province, Inner Mongolia, and Beijing, constituting approximately 30, 14, and 11% of the participants, respectively.

### 2.2 Outcome variables

The primary outcomes under investigation encompassed the length of hospital stay in days, total cost, and specific utilization categories. Presently, these hospitals operate within a pay-for-service framework, where the cost of hospitalization serves as an indicator of resource utilization during the clinical treatment of patients throughout their hospital stay. Furthermore, in the initial pages of hospitalization records, costs are itemized into distinct groups, including drug expenses, lab tests, medical imaging, medical treatment, surgery, anesthesia, blood costs, medical supplies and consumables, inpatient bed care, nursing services, physician and other miscellaneous services. For the purposes of this study, costs associated with surgical procedures—encompassing surgery, anesthesia, and blood-related services—were consolidated due to the relatively minor contribution of the latter two items to the total cost. Similarly, physician and other miscellaneous services were amalgamated for the same rationale.

### 2.3 Independent variables

In this study, we employed a conceptual framework based on Andersen’s health behavior model to assess eight variables, categorized as prerequisite, enabling, and demand variables, in the context of health services utilization among hospitalized patients with human brucellosis. Prerequisite variables include age, gender, and ethnicity. Enabling variables encompass the type of health insurance. Lastly, demand variables include different infection sites, conditions at admission, the Elixhauser comorbidity index, and whether the patient underwent surgery.

Specifically, the influencing factors were as follows: age, categorized into six groups—1–17 years old, 18–29 years old, 30–44 years old, 45–59 years old, 60–69 years old, and 70 years old and above; gender; ethnicity, categorized as Han and other ethnicities; and type of health insurance, divided into four groups—local health insurance, cross-provincial settlement, out-of-pocket, and others. Local health insurance includes basic medical insurance for urban employees, urban resident medical insurance, and new rural cooperative medical insurance that covers local citizens incorporated into basic medical insurance for urban and rural residents through reform. Cross-provincial settlement allows any patient enrolled in the public medical insurance system to be reimbursed for medical and inpatient expenditures, irrespective of the province where they received treatment. Out-of-pocket and other insurance mainly include government medical insurance. Cases grouped under the need for surgery considered whether the patient underwent surgery or not; infection sites, grouped into four categories—overall body, spine, joints, and other sites (including nerve, brain, and liver). According to standard rules, the condition at admission was classified into three categories: critical, urgent, and mild, identified based on the physician’s knowledge. The comorbidity index, with 31 categories of comorbidity defined by the Elixhauser comorbidity index (8), was determined by the ICD-10 and reduced to categorical variables: less than or equal to 0, greater than or equal to 1, greater than or equal to 5, and greater than or equal to 10.

### 2.4 Statistical analysis

Outcome variables and covariables were statistically described, with participants categorized by sex, age, ethnicity, type of health insurance, condition at admission, comorbidity index, whether surgery was performed, and site of infection. Intergroup comparisons were conducted to examine differences. For length of stay (LOS) and cost, due to right-skewed distribution, intergroup comparison utilized the rank sum test. The statistical significance level was set at 0.05.

In this study, multiple linear regression analysis was performed to investigate factors influencing the length of stay and costs of hospitalization in human brucellosis cases, where the independent variables mentioned above were all included. Due to the right-skewed distribution of LOS and costs, logarithm transformations were applied to these two outcome variables in the multiple linear regression analysis. Additionally, to mitigate the impact of year-to-year variations in costs, the year of admission was controlled in a multiple linear regression model. Costs were adjusted using the consumer price index (CPI), with the costs of 2023 set as the reference year (index value of 100). All data processing and statistical analysis were conducted using Python 3.6 and R 4.2.2.

## 3 Results

### 3.1 Hospital stays and total cost

A comparative analysis was conducted on both the hospitalization duration and total costs across various groups categorized by eight covariables. Table 1 displays the number and proportion (in brackets) of patients in each group, along with the median [IQR] Length of Stay (LOS), costs, and *p*-values.

**Table 1:** Duration of hospital stays and corresponding total cost across different grouping variables.

		*N* (proportion)	Length of stay (median [IQR]) days	Total cost (median [IQR]) 10 k¥
Sex	Male	345 (73.88)	12.79 [7.02, 19.69]	1.59 [0.78, 5.68]
	Female	122 (26.12)	12.91 [7.69, 21.13]	1.72 [0.92, 6.11]
	p		0.63	0.412
Age	1–17	15 (3.21)	11.13 [6.63, 19.17]	0.84 [0.56, 1.36]
	18–29	53 (11.35)	8.73 [6.03, 15.78]	0.88 [0.59, 1.99]
	30–44	67 (14.35)	10.22 [6.88, 16.88]	1.33 [0.72, 3.77]
	45–59	207 (44.33)	12.96 [7.89, 20.79]	1.73 [0.85, 6.48]
	60–69	104 (22.27)	14.81 [7.83, 21.20]	1.81 [1.05, 8.01]
	> = 70	21 (4.50)	15.94 [12.79, 19.94]	3.87 [1.79, 8.03]
	p		0.058	<0.001
Ethnicity	Han	432 (92.51)	12.96 [7.18, 19.90]	1.65 [0.80, 5.90]
	Others	35 (7.49)	9.04 [6.52, 18.73]	1.33 [0.75, 2.39]
	*p*		0.228	0.179
Insurance	Local-site	111 (23.77)	9.21 [6.02, 17.78]	1.23 [0.71, 2.53]
	Cross-provincial settlement	205 (43.90)	13.99 [8.73, 21.92]	1.87 [0.98, 7.55]
	Out-of-pocket	126 (26.98)	13.35 [6.93, 18.78]	1.74 [0.87, 4.26]
	Others	25 (5.35)	7.70 [5.09, 13.32]	0.74 [0.44, 1.26]
	*p*		<0.001	<0.001
Condition at admission	Mild	350 (74.95)	12.95 [7.21, 19.88]	1.59 [0.79, 6.12]
	Others	117 (25.05)	11.68 [6.91, 19.68]	1.66 [0.84, 3.71]
	*p*		0.61	0.96
Elixhauser comorbidity index	1	313 (67.02)	12.94 [7.21, 20.01]	1.57 [0.78, 6.44]
	2	80 (17.13)	10.73 [6.02, 18.68]	1.48 [0.79, 2.76]
	3	16 (3.43)	11.28 [7.67, 19.21]	1.74 [1.11, 5.14]
	4	58 (12.42)	13.36 [8.78, 19.97]	1.83 [1.11, 4.29]
	*p*		0.438	0.265
Surgery	No	326 (69.81)	9.14 [6.01, 14.77]	1.13 [0.67, 1.87]
	Yes	141 (30.19)	20.70 [15.01, 24.21]	7.49 [5.68, 9.40]
	*p*		<0.001	<0.001
Infection site	Body	299 (64.03)	10.02 [6.08, 15.96]	1.24 [0.72, 2.37]
	Spines	96 (20.56)	18.80 [13.53, 23.76]	7.23 [1.86, 9.51]
	Joints	37 (7.92)	14.88 [9.59, 21.72]	2.15 [1.12, 6.80]
	Others	35 (7.49)	13.22 [7.02, 17.33]	1.74 [0.79, 2.56]
	*p*		<0.001	<0.001
Total		467 (100)	12.80 [7.08, 19.76]	1.63 [0.80, 5.73]

As illustrated in Table 1, human brucellosis exhibited a higher prevalence among males, constituting 73.88% of the total population. However, there were no significant differences in LOS or total costs between the male and female groups. Patient ages ranged from 1 to 78 years, with a median age of 51 years. The majority fell within the 45 to 59 years age bracket, representing 44.33% of the population. Total costs increased with age, showing significant differences among the six age groups. Specifically, the median hospitalization expenses for patients aged over 70 years was ¥38,700, approximately 4.6 times higher than that of patients aged between 1 and 17 years, and 2.2 times higher compared to those in the 45 to 59 year-old age bracket. However, hospitalization days did not significantly differ across age groups. Han ethnicity accounted for over 92% of the study population, yet LOS and total costs showed no differences between ethnic groups.

Patients with cross-provincial settlement had the highest proportion, exhibiting the longest median hospital stay of 13.99 days and the highest total costs of ¥18,700, followed by out-of-pocket, local-site, and other groups, with a median LOS of 13.35, 9.21 and 7.7 days respectively, and total cost of ¥17,400, ¥12,300 and ¥7,400, respectively. Significant differences in both LOS and total costs were observed among the four insurance groups. Regarding admission condition, a substantial portion of the population (74.95%) had mild conditions. In contrast, critical patients constituted 25.05%, with no significant differences in hospitalization days or total costs between the two groups. The majority of patients (67.02%) had an Elixhauser comorbidity index of level 1, with no differences in hospitalization days or total costs among the different index groups.

Approximately 30% of all patients required surgery, demonstrating significantly higher LOS and total costs than the non-surgical group. Specifically, patients who underwent surgery had a median LOS of 20.70 days and hospital expenses of ¥74,900. In contrast, the non-surgical group experienced a median stay of 9.14 days and incurred costs of ¥11,300. In terms of infection sites, body infections constituted 64% of human brucellosis cases, with proportions for spines, joints, and other sites at 20.56, 7.92, and 7.49%, respectively. In terms of median stay and total costs, spine infections topped the list with an average duration of 18.8 days and a cost of ¥72,300. Joint infections followed closely with a median stay of 14.88 days and a cost of ¥21,500. Other sites of infection had a median stay of 13.22 days and a cost of ¥17,400, while body infections had the lowest median stay of 10.02 days and a cost of ¥12,400. The total cost of spine infections was approximately six times higher than that of body infections, with significant differences in both stay and costs among the four infection sites.

In summary, firstly, both hospitalization duration and total costs exhibited significant differences among groups based on insurance status, surgery status, and infection site. Secondly, there were no significant differences in hospitalization duration or total costs among groups based on sex, ethnicity, admission condition, or Elixhauser comorbidity index. Thirdly, while hospitalization days did not significantly differ among age groups, total costs did show significant differences across these groups.

### 3.2 Hospitalization utilization categories

Table 2 presents a breakdown of total costs for the 467 patients in this study, totaling approximately 15.5 million yuan. The top five utilization categories, in descending order, were medical supplies and consumables of 6.07 million yuan, drugs of 3.95 million yuan, lab tests of 2.34 million yuan, surgical procedures (surgery, anesthesia, and blood) of 0.92 million yuan, and medical imaging costs of 0.83 million yuan, accounting for 39.13, 25.47, 15.07, 5.97, and 5.36% of the total costs, respectively. The total cost of medical supplies and consumables was approximately 1.5 times that of drugs. However, in terms of the median cost, the ranking of utilization categories changed. The top five cost items were drugs, lab tests, medical imaging, medical supplies and consumables, and inpatient bed care costs. Additionally, the median cost of drugs was approximately 6 times that of medical supplies and consumables. Notably, surgical procedures costs recorded a zero median.

**Table 2:** Characteristics of various utilization categories in 467 inpatient cases.

Utilizations	Overall (10 k¥)	%	Median [IQR]
Drug	394.81	25.47	0.56 [0.17,1.22]
Lab test	233.58	15.07	0.43 [0.29,0.63]
Medical imaging	83.10	5.36	0.14 [0.06,0.23]
Medical treatment	42.72	2.76	0.02 [0.00,0.11]
Surgical procedures	92.47	5.97	0.00 [0.00,0.25]
Medical supplies and consumables	606.64	39.13	0.09 [0.04,1.58]
Inpatient bed care	43.01	2.77	0.07 [0.04,0.11]
Nursing services	13.78	0.89	0.02 [0.01,0.04]
Others services	40.48	2.61	0.03 [0.00,0.15]
Total cost	1550.21	100.00	1.63 [0.80,5.73]

We conducted a comparison of the median of each charge type item among eight covariable groups, and the rank sum test results are provided in the Supplementary material. Drug costs significantly differed among age, insurance, surgery, and infection site groups. Lab tests, medical treatment, and nursing services costs showed significant differences based on insurance, Elixhauser comorbidity index, surgery, and infection site. For medical imaging costs and surgical procedures costs, significant differences were observed among groups based on age, insurance, Elixhauser comorbidity index, surgery, and infection site. Factors related to medical supplies and consumable costs were the same as those related to surgical procedures costs, except for the Elixhauser comorbidity index, where medical supplies and consumable costs did not exhibit a difference. Unlike other utilization categories, inpatient bed care and other service costs were not significantly different among insurance groups. Additionally, inpatient bed care costs were the only item that did not differ based on age, but they were the only ones that significantly differed among groups based on the condition at admission.

In summary, all utilization categories exhibited significant differences based on whether surgery was performed and the infection site groups, which were also associated with total cost and length of stay. Furthermore, all charge types, except for inpatient bed care and other services costs, significantly differed across insurance types.

### 3.3 Regression analysis of LOS and total cost

Table 3 provides multilinear regression results for hospital stays and total costs. Notably, patients admitted with mild conditions and those with body infections exhibited a shorter LOS compared to the reference group. Conversely, patients with an Elixhauser comorbidity index of level 2 or 4, as well as those who underwent surgery, experienced a longer LOS than the reference group. For total costs, individuals under the age of 18, those with mild conditions at admission, alternative insurance types, and those with joint and body infections demonstrated lower costs compared to the reference group. Conversely, patients with an Elixhauser comorbidity index of level 2 or 4, and those who underwent surgery, incurred higher costs.

**Table 3:** Multilinear regression coefficients for length of stay (LOS) and total cost among inpatients with brucellosis.

Variables		LOS	Total cost
Sex	Female	Reference	Reference
	Male	−0.0254 (0.0643)	−0.0271 (0.0805)
Age	18–29	Reference	Reference
	1–17	0.187 (0.186)	−0.427* (0.233)
	30–44	0.0200 (0.122)	0.0140 (0.153)
	45–59	0.0266 (0.111)	−0.00112 (0.139)
	60–69	−0.0131 (0.122)	0.00520 (0.152)
	> = 70	−0.0624 (0.167)	0.105 (0.209)
Ethnicity	Others	Reference	Reference
	Han	0.0420 (0.108)	0.0929 (0.136)
Condition at admission	Others	Reference	Reference
	Mild	−0.112* (0.0679)	−0.295*** (0.0850)
Insurance	Local-site	Reference	Reference
	Cross province settlement	0.0394 (0.0822)	0.0434 (0.103)
	Out-of-pocket	−0.135 (0.0942)	−0.101 (0.118)
	Others	0.00838 (0.155)	−0.464** (0.194)
Elixhauser comorbidity index	1	Reference	Reference
	2	0.135* (0.0790)	0.311*** (0.0989)
	3	0.248 (0.159)	0.385* (0.199)
	4	0.270*** (0.0903)	0.562*** (0.113)
Infection site	Spine	Reference	Reference
	Joint	−0.0504 (0.127)	−0.430*** (0.159)
	Others	−0.134 (0.140)	−0.269 (0.175)
	Body	−0.176* (0.0955)	−0.304** (0.120)
Surgery	No	Reference	Reference
	Yes	0.567*** (0.0864)	1.469*** (0.108)
Admission year	2013	Reference	Reference
	2014	−0.118 (0.122)	−0.130 (0.153)
	2015	−0.201 (0.126)	−0.0998 (0.157)
	2016	−0.193 (0.126)	0.0812 (0.158)
	2017	−0.0223 (0.139)	0.310* (0.174)
	2018	−0.118 (0.151)	0.246 (0.189)
	2019	−0.245* (0.141)	0.127 (0.176)
	2020	0.321 (0.212)	0.477* (0.265)
	2021	−0.139 (0.135)	0.138 (0.170)
	2022	−0.0921 (0.142)	0.204 (0.178)
	2023	−0.380*** (0.145)	−0.0816 (0.181)
Constant		2.724*** (0.262)	9.161*** (0.329)
Observations		467	467
R-squared		0.338	0.600

Standard errors in parentheses.

*** *p* < 0.01, ** *p* < 0.05, * *p* < 0.1.

In summary, the condition at admission, Elixhauser comorbidity index, infection site, and surgery were associated with both the length of stay and total cost. Additionally, age and insurance type had an impact on the total cost.

Multiple linear regression analysis was also conducted on each utilization category, and the results are detailed in the Supplementary materials. The analysis revealed that gender was not associated with any utilization category, while age only impacted surgical procedures costs and inpatient bed care costs. Ethnicity was associated with all utilization categories except for drugs, medical imaging, and other services costs. The patient’s condition at admission influenced drug, medical treatment, and other services costs. Insurance type was related to lab tests, medical imaging, medical treatment and inpatient bed care costs. The Elixhauser comorbidity index presented an association with all utilization categories except for medical imaging and other services costs. The infection site influenced drug, surgical procedures, and medical supplies and consumable costs. Whether surgery was performed had an impact on all utilization categories except for other services costs.

## 4 Discussion

Human brucellosis holds paramount significance in contemporary public health discourse. Although the disease has a low mortality rate and usually respond to the adequate antibiotic therapy, only 35% of brucellosis patients that seek medical attention for the first time are correctly diagnosed due to unspecific symptoms (9). Additionally, human brucellosis is associated with high healthcare utilization and hospitalization costs, which has also been proved in other studies (10). Therefore, this study is imperative for developing targeted interventions and reducing its burden on society.

The existing body of research on human brucellosis in China has predominantly focused on its clinical manifestations (11, 12), various complications (12, 13), treatment (14, 15), epidemiological characteristics (16–18), shedding light on the clinical understanding of the disease. Although there are some studies investigated the healthcare expenditure associated with human brucellosis, including those that estimated treatment costs ranging from 9 euros in Tanzania, 200 euros in Morocco, 650 euros in Algeria (19), and up to $1,000 each infected individual in Argentina (20), a significant gap remains in the literature concerning a thorough exploration of the cost and length of stay (LOS) implications of this disease. This absence of in-depth analysis hinders our comprehensive understanding of the economic burden of human brucellosis and the critical need for priority setting (21–24). Both cost and LOS provide decision-makers with a better understanding of the impact of some of the diseases and the research effort that goes into them (25). Our study filled this gap by offering a detailed analysis of inpatients’ utilization of healthcare resources, reflected by LOS, total cost, and various utilization categories of healthcare expenditure.

Based on this study, it can be concluded that both LOS and total cost were significantly different concerning insurance, surgery or not, and infection sites. For the utilization categories, they all demonstrated significant differences between groups classified by having surgery or not and infection sites. Additionally, through multiple linear regression analysis, we found that the condition at admission, Elixhauser comorbidity index, infection site, and whether surgery was performed all influenced both hospital stays and total cost. In addition, age and insurance type were associated with total costs.

Concerning utilization categories, the total medical supplies and consumables cost amounted to over six million yuan, accounting for approximately 40% of the total cost, and exceeded the costs of drugs, lab tests, and medical imaging. However, the median medical supplies and consumable cost was lower than these three costs and ranked fourth among all utilization categories. This is because the medical supplies and consumables cost for all patients in this study had a wide range, but a small proportion of participants contributed the majority of the expense. Specifically, the minimum and maximum medical supplies and consumable costs were 0 and 91.31 thousand yuan, respectively. The sum of medical supplies and consumables costs between the third and fourth quartiles was 5.65 million yuan, accounting for 94.2% of the total medical supplies and consumables cost. And it was observed in this study patients with high medical supplies and consumable costs often undergo orthopaedic surgery, including thoracic fusion, lumbosacral fusion, excision of lumbar lesions, and thoracic joint fixation surgery. As reported in (26), Brucellar spondylodiscitis is the most prevalent and significant osteoarticular presentation of human Brucellosis, and involves multiple structures including vertebral bodies, intervertebral disc, and paraspinal structures. Certain patients may require surgical treatment if spinal instability, abscess formation or intractable pain are observed.

Compared with medical supplies and consumables cost, drug cost had fewer outliers, and the sum between the third and fourth quartile amounted to 2.45 million yuan, contributing 61.9% of the total drug cost. In this study, nonantibiotic and anti-infective drugs were commonly used, with anti-tuberculosis *mycobacterium leprae* drugs and quinolone antibiotic drugs accounting for 55 and 26%, respectively. Specifically, rifampicin, doxycycline hydrochloride, ethambutol hydrochloride, chlorhexidine acetate solution, and compound sulfamethoxazole were the top five most frequently used drugs for all patients.

Brucellosis can involve the human body systemically (27). In this study, we observed that patients were admitted to various departments, including orthopedics, neurology, respiratory, hematology, hepatic, and infectious diseases departments. Motivated by this observation, we conducted a detailed analysis of the significant differences among infection sites for each charge type. The results demonstrated significant differences in infection sites among each charge type. Specifically, spine infection group had the highest hospital stay, total costs and all utilization categories except for examination cost. This is because spondylodiscitis is the most severe form of osteoarticular involvement by brucellosis, and can have single or multifocal involvement (27). In addition, surgery rates differed significantly between different infection groups. In particular, the spine infection site achieved the highest surgery ratio of 72.92%, exceeding more than 40% of the average. In contrast, the body, joints, and other infection sites had surgery rates of 16.7, 54.1, and 2.9%, respectively.

In this study, we identified a strong correlation between the infection site of human brucellosis and all utilization categories examined, emphasizing the importance of considering this factor when assessing the economic burden of human brucellosis. Notably, few previous studies in China have specifically investigated the cost implications associated with the infection site (28, 29), highlighting a critical knowledge gap. This study contributes to filling this void by providing detailed insights into how the location of infection impacts various cost components. Given the significant influence of infection site on the financial burden shouldered by patients, it is imperative to consider targeted measures to alleviate this burden (30).

Physicians’ decisions play a key role in overall health care spending and quality, and they are encouraged to exercise wise stewardship of resources (31). Based on the findings of this study, physicians are suggested to take into account the condition at admission, Elixhauser comorbidity index, infection site, and whether surgery was performed to estimate the hospital stays and total costs of inpatients diagnosed with human brucellosis. In addition, understanding the various influencing factors benefits both hospital managers in optimizing resource utilization and patients in receiving more tailored and effective care. To mitigate the impact of human brucellosis, healthcare policies should prioritize preventive strategies, early detection, and personalized treatment protocols, which may lead to decreased hospitalization periods and subsequent healthcare costs (32). Additionally, raising awareness about transmission routes and promoting safety measures in high-risk occupations, such as livestock farming and veterinary work, can play a pivotal role in preventing human brucellosis and mitigating its economic consequences on affected individuals and the healthcare system (33). Our analysis has several limitations. Firstly, this study collected patient information from eight large tertiary hospitals, so the results may not be widely applicable to community hospitals due to differences in facilities, scale, and the quality of medical services between tertiary hospitals and community hospitals. Secondly, the retrospective nature of our study cannot guarantee the collection of all relevant information tailored specifically to the healthcare utilization of patients with human brucellosis. For instance, socioeconomic status may empirically have an impact on the hospital stays and costs, but our analysis did not take into account this potential confounding factor due to the unavailability of relevant data. In future research, we aim to comprehensively analyze the economic burden associated with human brucellosis, encompassing both outpatient populations and community hospitals. By broadening our scope to include these additional groups, we aim to further enhance the reliability and generalizability of our conclusions.

## Data availability statement

The original contributions presented in the study are included in the article/Supplementary material, further inquiries can be directed to the corresponding authors.

## Ethics statement

The studies involving humans were approved by Medical Ethics Committee of Chinese PLA General Hospital. The studies were conducted in accordance with the local legislation and institutional requirements. Written informed consent for participation was not required from the participants or the participants’ legal guardians/next of kin in accordance with the national legislation and institutional requirements.

## Author contributions

XM: Conceptualization, Investigation, Methodology, Writing – original draft, Writing – review & editing. WW: Formal analysis, Investigation, Software, Writing – original draft, Writing – review & editing. QW: Writing – original draft, Writing – review & editing. CZ: Data curation, Writing – review & editing. JL: Data curation, Writing – review & editing. HB: Data curation, Writing – review & editing. TZ: Methodology, Writing – review & editing. LinL: Methodology, Software, Writing – review & editing. LihL: Conceptualization, Funding acquisition, Project administration, Supervision, Writing – review & editing.
